# Modifiable Psychosocial Predictors of Healthy Cognitive Aging in Daily Life

**DOI:** 10.1093/geroni/igaf122.181

**Published:** 2025-12-31

**Authors:** Jeremy Hamm, Eric Cerino

## Abstract

Healthy cognitive aging is shaped in part by modifiable (intervenable) psychosocial factors. However, little remains known about how these dynamic processes operate in daily life, and the extent to which they are implicated in day-to-day changes in cognitive functioning. This symposium addresses the role of daily beliefs and appraisals (perceived control, stressor severity), emotions (affect valence and intensity), and strategies (goal engagement) as predictors of cognitive health and functioning in daily life. Klepacz et al. investigate how, across the adult lifespan, individual differences in perceived control predict day-to-day variability in cognitive functioning, a sensitive indicator of early changes in cognition that confers increased risk of later impairment. Cerino et al. use data from the National Study of Daily Experiences to document how interpersonal stressor control is linked to memory lapses and associated irritation in daily life, especially in older adulthood. Richards et al. use ecological momentary assessment (EMA) data to identify the contextual circumstances under which stress severity predicts moment-to-moment working memory performance. Castle et al. employ EMA data to examine how affect pleasantness, unpleasantness, and intensity are associated with momentary processing speed and working memory. Finally, Hamm et al. use measurement-burst data to document how the investment of time and persistent effort into valued goals (goal engagement) is linked to daily episodic memory and executive functioning. This symposium contributes to a more comprehensive understanding of how healthy cognitive aging may be shaped by dynamic moment-to-moment and day-to-day changes in modifiable beliefs, emotions, and strategies across the adult lifespan.

